# Supplementary material for: Mass Spectrometry Study about In Vitro and In Vivo Reaction between Metformin and Glucose: A Preliminary Investigation on Alternative Biological Behavior
Source: Int J Mol Sci. 2023 Dec 22;25(1):180. doi: 10.3390/ijms25010180 (PMC10779030; doi:10.3390/ijms25010180)
Supplement: Supplementary file 1 [file ijms-25-00180-s001.zip › ijms-2764932-supplementary.pdf]

Mass Spectrometry Study about In Vitro and In Vivo Reaction between Metformin and Glucose: A Preliminary Investigation on Alternative Biological Behavior

Gianluca Bartolucci [1,*], Marco Pallecchi [1], Laura Braconi [1], Silvia Dei [1], Elisabetta Teodori [1], Annunziata Lapolla [2], Giovanni Sartore [2], Pietro Traldi [3,*]

[1] Dipartimento di Neuroscienze, Psicologia, Area del Farmaco e Salute del Bambino (NEUROFARBA), Università di Firenze, 50100 Firenze, (Italy); gianluca.bartolucci@unifi.it (G.B.); marco.pallecchi@unifi.it (M.P.); laura.braconi@unifi.it (L.B.); silvia.dei@unifi.it (S.D.); elisabetta.teodori@unifi.it (E.T.).

[2] Dipartimento di Medicina, Università di Padova, 35100 Padova, (Italy); annunziata.lapolla@unipd.it (A.L.); g.sartore@unipd.it (G.S.).

[3] Istituto di Ricerca Pediatrica Città della Speranza, 35100 Padova, (Italy); p.traldi@irpcds.org (P.T.).

* Correspondence: p.traldi@irpcds.org and gianluca.bartolucci@unifi.it

Supplementary Materials

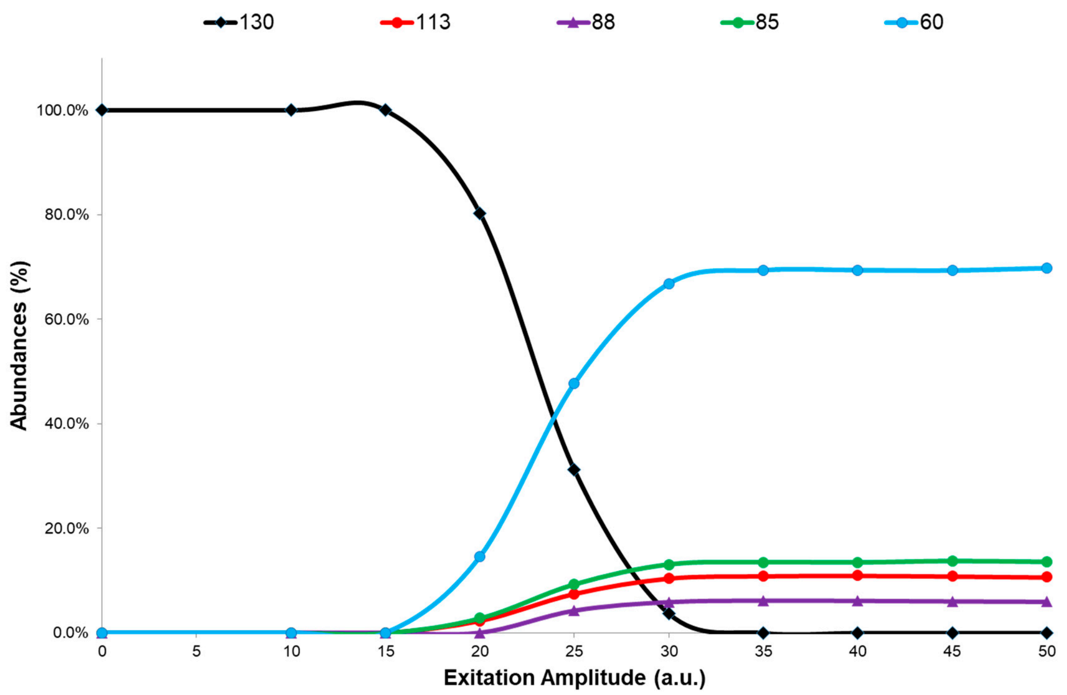

Figure S1. Collision breakdown curves of $[M+H]^+$ of metformin (m/z 130) at different excitation amplitudes.

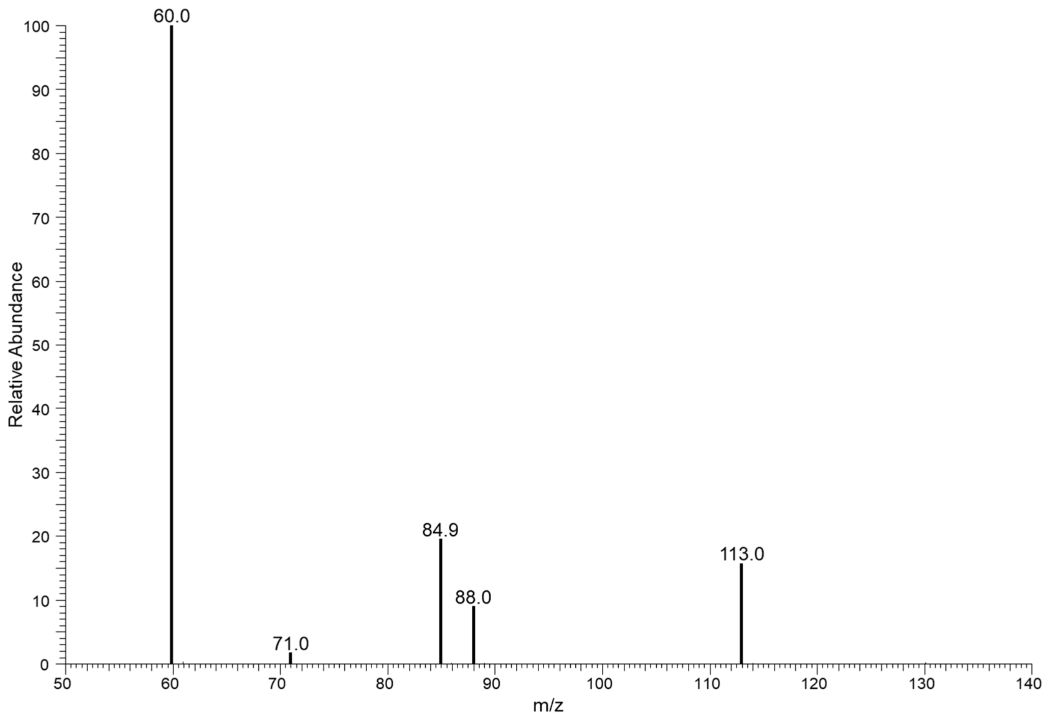

Figure S2. MS/MS spectrum at its ExA$_{max}$ of metformin.

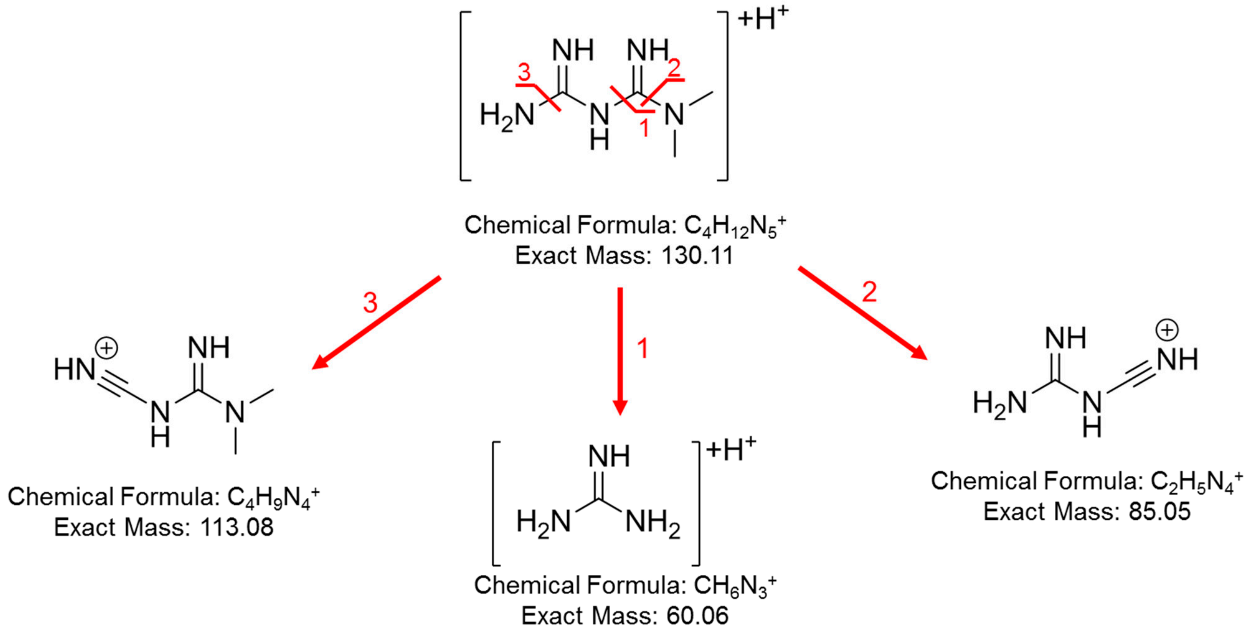

Figure S3. Proposed MS/MS fragmentation pathway for the metformin.

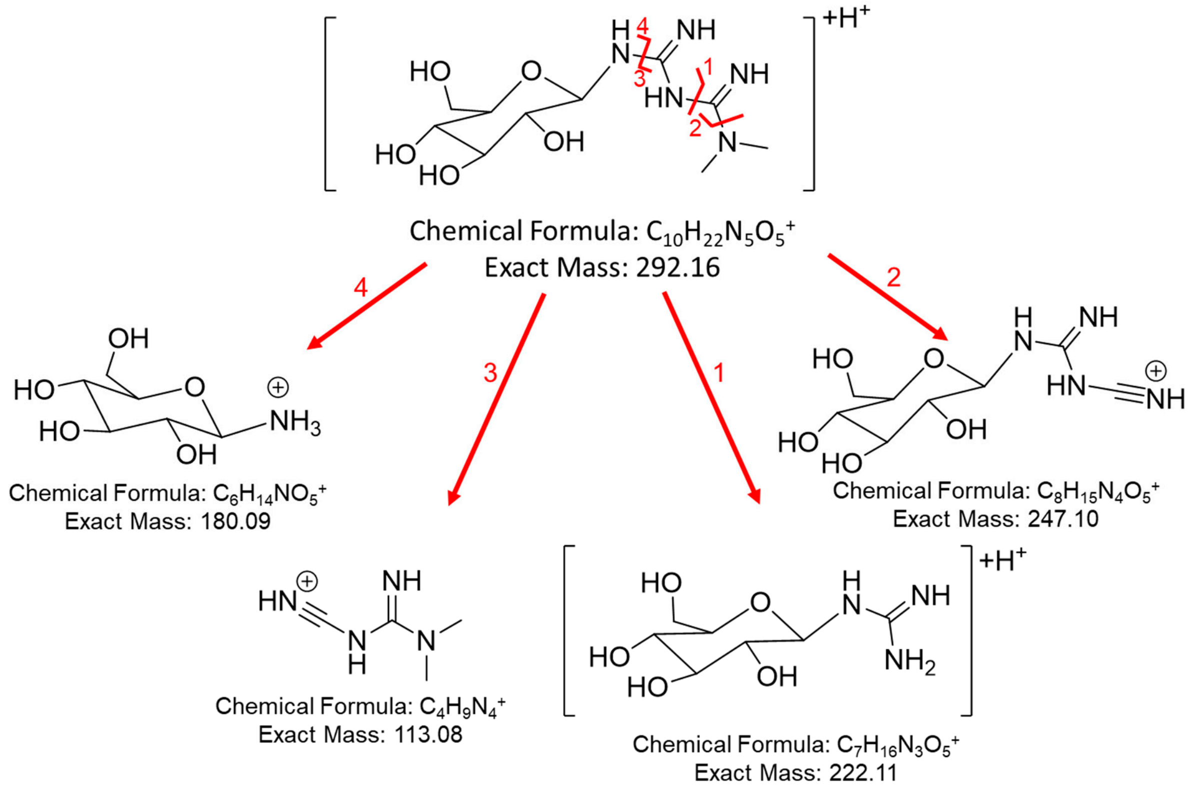

Figure S4. Proposed MS/MS fragmentation pathway for the metformose.

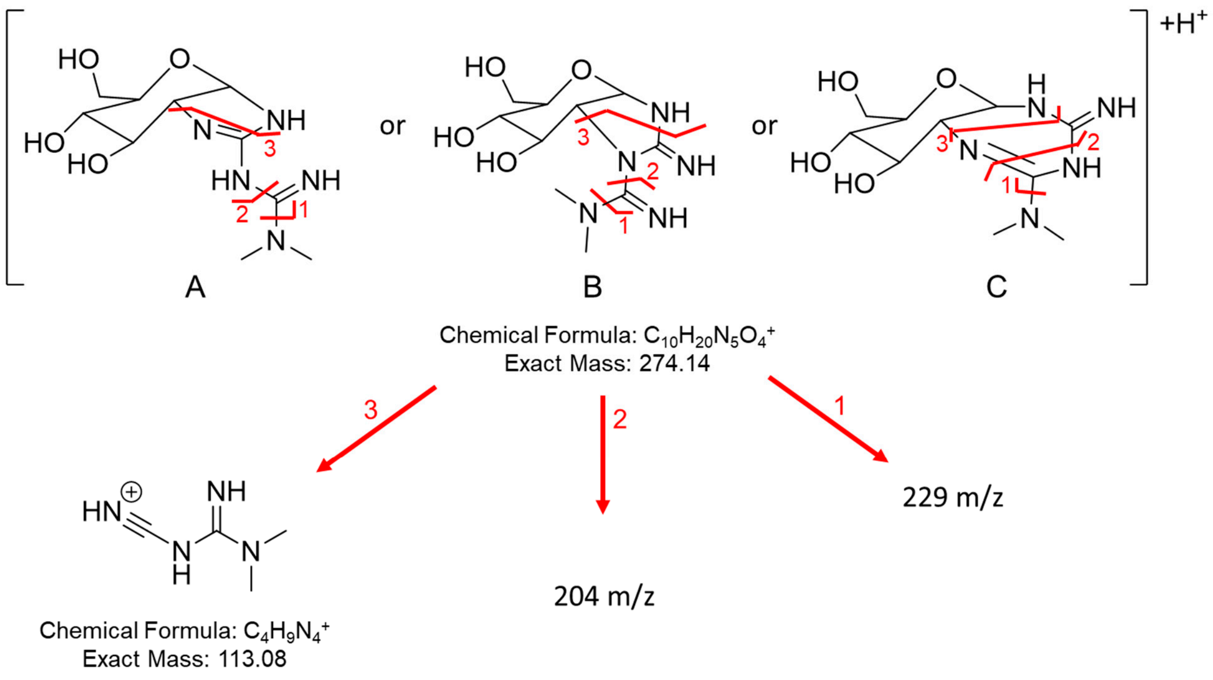

Figure S5. Proposed MS/MS fragmentation pathway for the DHmetformose.
